# Association Between Receipt of Unemployment Insurance and Food Insecurity Among People Who Lost Employment During the COVID-19 Pandemic in the United States

**DOI:** 10.1001/jamanetworkopen.2020.35884

**Published:** 2021-01-29

**Authors:** Julia Raifman, Jacob Bor, Atheendar Venkataramani

**Affiliations:** 1 Department of Health Law, Policy, and Management, Boston University School of Public Health, Boston, Massachusetts; 2 Department of Global Health, Boston University School of Public Health, Boston, Massachusetts; 3 Leonard Davidson Institute for Health Economics, University of Pennsylvania, Philadelphia; 4 Medical Ethics and Health Policy, University of Pennsylvania, Philadelphia

## Abstract

**Importance:**

More than 50 million US residents have lost work during the coronavirus disease 2019 (COVID-19) pandemic, and food insecurity has increased.

**Objective:**

To evaluate the association between receipt of unemployment insurance, including a $600/wk federal supplement between April and July, and food insecurity among people who lost their jobs during the COVID-19 pandemic.

**Design, Setting, and Participants:**

This cohort study used difference-in-differences analysis of longitudinal data from a nationally representative sample of US adults residing in low- and middle-income households (ie, <$75 000 annual income) who lost work during the COVID-19 pandemic. Data were from 15 waves of the Understanding Coronavirus in America study (conducted April 1 to November 11, 2020).

**Exposure:**

Receipt of unemployment insurance benefits.

**Main Outcomes and Measures:**

Food insecurity and eating less due to financial constraints, assessed every 2 weeks by self-report.

**Results:**

Of 2319 adults living in households earning less than $75 000 annually and employed in February 2020, 1119 (48.3%) experienced unemployment during the COVID-19 pandemic and made up our main sample (588 [53.6%] White individuals; mean [SD] age 45 [15] years; 732 [65.4%] women). Of those who lost employment, 415 (37.1%) reported food insecurity and 437 (39.1%) reported eating less due to financial constraints in 1 or more waves of the study. Among people who lost work, receipt of unemployment insurance was associated with a 4.3 (95% CI, 1.8-6.9) percentage point decrease in food insecurity (a 35.0% relative reduction) and a 5.7 (95% CI, 3.0-8.4) percentage point decrease in eating less due to financial constraints (a 47.8% relative reduction). Decreases in food insecurity were larger with the $600/wk supplement and for individuals who were receiving larger amounts of unemployment insurance.

**Conclusions and Relevance:**

In this US national cohort study, receiving unemployment insurance was associated with large reductions in food insecurity among people who lost employment during the COVID-19 pandemic. The $600/wk federal supplement and larger amounts of unemployment insurance were associated with larger reductions in food insecurity.

## Key Points

**Question:**

Was the receipt of unemployment insurance and a $600/wk federal supplement to unemployment insurance associated with reduced food insecurity among people in low- and middle-income households who lost work during the coronavirus disease 2019 (COVID-19) pandemic?

**Findings:**

In this cohort study of 1119 adults who lost work during the COVID-19 pandemic, unemployment insurance was associated with a 35% relative decline in food insecurity and a 48% relative decline in eating less due to financial constraints. The $600/wk federal supplement was associated with additional reductions in food insecurity.

**Meaning:**

These findings suggest that expanding the amount and duration of unemployment insurance may be an effective approach to reducing food insecurity.

## Teaser

This cohort study evaluates the association between receipt of unemployment insurance, including a $600/wk federal supplement between April and July, and food insecurity among US residents who lost their jobs during the coronavirus disease 2019 (COVID-19) pandemic.

## Introduction

Since the coronavirus disease 2019 (COVID-19) pandemic began, more than 54 million US residents have lost their jobs,^1^ with most of these individuals living in low-income households. The loss of earnings has made many US residents vulnerable to food insecurity,^2,3^ defined by the US Department of Agriculture as “limited or uncertain access to adequate food.” Food insecurity has been linked to worse general health and well-being^4,5,6^; depression, anxiety, and suicidal ideation^7^; interpersonal stress and challenges^6,8,9,10^; chronic disease^5^; and adverse child development outcomes.^4,11^ Food insecurity has more than doubled during the COVID-19 pandemic and increased even further in households with children.^12^

Unemployment insurance (UI) was expanded during the COVID-19 pandemic. The federal Coronavirus Aid, Relief, and Economic Security (CARES) Act authorized a $600/wk federal supplement to state UI benefits, which terminated at the end of July 2020. The CARES Act also expanded UI eligibility and duration of benefits until December 26, 2020. On December 27, 2020, Congress continued federal funding for expanded UI eligibility and duration and implemented a $300/wk federal UI supplement until March 14, 2021.

Historically, there is evidence that UI is associated with reduced food insecurity.^13^ The association between UI and food insecurity during the COVID-19 pandemic—a time of both unprecedented hardship and, from April to July, unprecedented generosity of UI benefits—has not been well established, although there is initial evidence that those who received UI reported lower food insecurity.^14^ We conducted a longitudinal, difference-in-differences analysis of the association between UI and food insecurity among people who lost their jobs between April and November 2020.

## Methods

### Data Source

We used data from the Understanding Coronavirus in America (UCA) study, collected by the University of Southern California Center for Economic and Social Research (CESR).^15^ The UCA is an extension of an ongoing internet-based, nationally representative longitudinal research survey. Beginning on April 1, 2020, CESR conducted UCA surveys with the full cohort every 2 weeks (eTable 1 in the Supplement). During each 2-week period, cohort members were invited to respond on a randomly selected day. We used data collected over 15 survey waves, between April 1 and November 11, 2020. The analyses of deidentified secondary data were considered non–human participant research and therefore exempt from institutional review board approval according to the Boston University Medical Center. Informed consent was obtained from all participants. We followed the Strengthening the Reporting of Observational Studies in Epidemiology (STROBE) reporting guideline for cohort studies.

### Study Sample

We restricted the sample to people who were living in households earning less than $75 000 in the last 12 months (ie, the income groups in which ≥20% people reported food insecurity in 1 or more survey waves) (eFigure 1 in the Supplement); who participated in at least 2 survey waves; who reported being employed in February 2020 (ie, prior to the COVID-19 pandemic); and who lost employment at some point during the April to November study period. We adopted the last sample restriction because we anticipated that trends in food insecurity would differ among those who remained employed.

Household income was based on self-report the first time a participant was surveyed about their household demographic information during the study period. We considered people to have been employed in February based on any response besides not employed or retired to the question, “Thinking back to February 2020, were you employed by the government, employed by a private company, employed by a nonprofit organization, self-employed, not employed, or retired?” Participants who lost their jobs during the study period were identified as those who responded no to the question, “Do you currently have a job?”

### Exposures

The primary exposure of interest was the start of UI benefits, a binary variable coded as 1 beginning in the first wave in which the respondent answered yes to the question “Have you received UI benefits in the past 14 days?” and as 1 thereafter. This question was coded 0 for individuals who reported currently having a job. In secondary analyses, we evaluated UI with and without the CARES supplement and in different amounts. In the first secondary analysis, we considered people to be receiving UI with the CARES supplement when they received UI before July 31 and did not report receiving less than $600/wk. We considered people to be receiving UI without the CARES supplement when they received UI after July 31 or reported receiving less than $600/wk. The second secondary exposure of interest was the amount of UI (ie, $1 to $300, $301 to $600, $601 to $900, $901 to $1200, $1201 to $1500, and ≥$1500), with all those not receiving UI coded as 0.

### Outcomes

Questions on food insecurity were from the Food Insecurity Experience Scale developed by the Food and Agricultural Organization of the United Nations.^16^ Food insecurity reflects being uncertain about having enough food and was binary based on the question, “In the past 7 days, were you worried you would run out of food because of a lack of money or other resources?” We coded yes responses as 1, no responses as 0, and unsure responses as missing. The second outcome of interest was whether respondents reported eating less due to financial constraints, which reflected reduced food quantity and was also binary based on the question, “In the past 7 days, did you eat less than you thought you should because of a lack of money or other resources?”

### Covariates

In all analyses, we included individual fixed effects to adjust for time-invariant individual characteristics. We included survey wave fixed effects to adjust for national secular trends in exposure to UI benefits and the outcomes of interest. We also adjusted for several time-varying, self-reported covariates, including receiving a federal stimulus payment, receiving Supplemental Nutrition Assistance Program (SNAP) benefits in the month prior to the survey, and employment status at the time of the survey. The CARES Act included a 1-time $1200 stimulus payment to each adult and $500 to each dependent child in low- and middle-income households. Receipt of the stimulus payment was coded as 1 after the first wave in which the respondent reported receiving economic stimulus funds and 0 otherwise. We coded SNAP benefits as 1 or 0 for each wave based on whether anyone in the household was reported to receive “Supplemental Nutrition Assistance Program (SNAP or Food Stamps).” SNAP eligibility was not broadly expanded during the pandemic, so there was little within-person variation in receipt of SNAP benefits during the study period that would not be captured by individual fixed effects. The inclusion of stimulus and SNAP benefits as covariates also accounts for receipt of other services that may be associated with both receipt of UI benefits and food insecurity. The inclusion of employment helps to restrict model comparisons to individuals who are unemployed and received UI vs individuals who are unemployed and did not. Because the UCA study only collected data on receipt of UI from people without current employment for the first 5 waves of the study, we coded UI as 0 for all people with employment in the latter 10 waves as well.

### Statistical Analysis

We first described the demographic characteristics and household composition of people in the sample and their reports of food insecurity and eating less during the study period. Race, ethnicity, and all other characteristics were based on participant self-report to Understanding America Study categories. We combined variables describing participant race and ethnicity and included these variables to document disparities shaped by structural racism.

We then used a difference-in-differences research design to compare changes in food insecurity and eating less before and after individuals received UI relative to changes in the same outcomes over time among individuals with no change in UI receipt during that period^17^ (eAppendix in the Supplement).^1^ We ran 3 separate models to evaluate any unemployment insurance, unemployment insurance and the CARES act $600/wk UI supplement, and UI amount. The validity of difference-in-difference methods rests on the assumption that those receiving UI in a given period would have had the same trends in food insecurity as those who did not receive UI or received it in different periods if UI recipients had not received UI.^17^

In addition to a standard difference-in-differences model, we estimated complementary event study models,^18,19^ in which we estimated the differences in the outcome associated with each time period before and after individuals reported first receiving UI, which differed for each individual. In this specification, the primary exposure variables of interest were indicator variables for 4-week periods relative to receiving the first UI payment. Individuals in the sample who never received UI were assigned 0 for all of the time period indicators.

The event study approach has 2 advantages for our research question. First, the flexible estimation strategy enables us to evaluate whether there were parallel trends in food insecurity prior to UI receipt, suggesting that parallel trends might have continued if those who received UI had not received it. Second, the event study specification allows assessment of treatment effects over time and helps to reduce bias when treatment timing varies.^18^

We used linear regression for all analyses due to evidence that logistic models can underestimate standard errors in the presence of fixed effects.^20^ Throughout, we clustered standard errors by individuals to account for serial correlation in the outcomes.^21^

We conducted 5 sensitivity analyses. First, we ran the main difference-in-difference models restricted to individuals who participated in the UCA survey at least once in each full month, April to October. Second, we ran the main difference-in-difference models with survey weights designed to make the sample nationally representative, a specification check.^22^ Third, we ran the main difference-in-differences models as logistic rather than linear models. Fourth, we restricted the analysis to those currently unemployed. Fifth, we ran the analysis in the subgroup of people living in households earning less than $20 000 per year. We also initially conducted analyses from April to July 2020 and later incorporated new data collected through November 2020.

Analyses were conducted with Stata version 15 (StataCorp). Statistical significance was set at *P* < .05, and all tests were 2-tailed.

## Results

### Sample Characteristics

There were a total of 7684 participants in the UCA. Of 7596 participants who reported whether they were employed in February, 4915 (64.7%) were employed. Of those who were employed in February, 2623 (56.9%) had a household income of less than $75 000, of whom 2550 (97.2%) responded to 2 or more UCA surveys and did not have missing data for covariates (eTable 1 in the Supplement). Participants who were younger, had lower-income, and were transgender individuals were more likely to be excluded due to missing covariates. Of those with complete data, 1119 participants (43.9%) reported being unemployed during at least 1 wave of the UCA between April 1 and November 11, 2020, and were the main sample for our analyses. Participants in this sample responded to a mean (SD) of 11.3 (4.1) of 15 UCA waves.

Of the 1119 people in the main sample, most (588 [53.6%]) were non-Hispanic White individuals, 265 (23.7%) were Hispanic individuals, 135 (12.1%) were non-Hispanic Black individuals, and the remainder identified as other races and ethnicities (Table 1). The mean (SD) age was 45 (15) years, and 732 (65.2%) were women. Overall, 126 (11.3%) reported being lesbian, gay, or bisexual, and 19 (1.7%) reported being transgender or nonbinary. A plurality of participants (497 [44.4%]) lived in households with 2 or more adults and no children, 268 (23.9%) lived alone, 307 (27.4%) lived in households of 2 or more adults with children, and 47 (4.2%) were single adults with children. A total of 282 participants (25.2%) lived in households earning less than $20 000, and 478 (42.7%) of participants in the sample received UI at some point during the study period. Those who received UI were similar to those who did not (Table 1).

**Table 1.** Characteristics of Participants Who Lost Work by Receipt of Unemployment Insurance

Characteristic^a^	No. (%)		*P* value
	Unemployment insurance (n = 478)	No unemployment insurance (n = 641)	
Race and ethnicity			
Non-Hispanic			
White	249 (52.1)	339 (52.9)	.79
Black	49 (10.3)	86 (13.4)	.11
American Indian or Alaska Native	5 (1.1)	8 (1.3)	.76
Asian	26 (5.4)	36 (5.6)	.90
Hawaiian or Pacific Islander	1 (0.2)	1 (0.2)	.84
Mixed race	26 (5.4)	28 (4.4)	.41
Hispanic	122 (25.5)	143 (22.3)	.21
Sex			
Women	299 (62.6)	433 (67.6)	.08
Men	179 (37.5)	208 (32.5)	.08
Income group, $			
<20 000	110 (23.0)	172 (26.8)	.14
20 000-29 999	81 (17.0)	95 (14.8)	.33
30 000-39 999	81 (17.0)	105 (16.4)	.80
40 000-59 999	137 (28.7)	156 (24.3)	.10
60 000-74 999	69 (14.4)	113 (17.6)	.15
Age group, y			
18-29	91 (19.0)	132 (20.6)	.52
30-39	121 (25.3)	130 (20.3)	.046
40-49	88 (18.4)	99 (15.4)	.19
50-59	102 (21.3)	127 (19.8)	.53
≥60	76 (15.9)	153 (23.9)	.001
Sexual orientation			
Heterosexual	424 (88.7)	569 (88.8)	.97
LGB	54 (11.3)	72 (11.2)	.97
Gender identity			
Cisgender	469 (98.1)	631 (98.4)	.68
Transgender or nonbinary	9 (1.9)	10 (1.6)	.68
Adults in households with no children, No.			
1	105 (22.0)	163 (25.4)	.18
2	226 (47.3)	271 (42.3)	.10
Adults in households with children, No.			
1	21 (4.4)	26 (4.1)	.78
≥2	126 (26.4)	181 (28.2)	.50

Abbreviation: LGB, lesbian, gay, or bisexual.

^a^ Characteristics are based on the first observation for each participant in the sample. All observations are from individuals who participated in at least 2 waves of the UCA survey. Numbers and percentages represent the full sample and are unweighted.

The proportion of those employed in February and earning less than $75 000 who reported not having a job in any given wave of the survey ranged from a high of 31.1% (599 of 1925) during the wave 2 (April 15 to May 12) survey to a low of 22.5% (434 of 1932) during the wave 13 (September 2-30) survey. Participants who were Hispanic individuals; were Black individuals; had lower income; were lesbian, gay, or bisexual individuals; and were transgender or nonbinary individuals were more likely to report unemployment (eTable 2 in the Supplement).

### Food Insecurity

Overall, 415 participants (37.1%) reported food insecurity and 437 (39.1%) reported eating less due to financial constraints during at least 1 wave of the survey (Table 2). During the April 1 to November 11 study period, the mean (SD) proportion of participants reporting food insecurity across all observations was 12.3% (32.9%) and the mean (SD) proportion of participants reporting eating less was 11.9% (32.4%). The greatest level of food insecurity was in the first wave of the survey (April 1 to April 28), when 165 of 743 participants (22.2%) reported food insecurity. Food insecurity was lowest in the second-to-last wave of the survey (September 30 to October 26), at 8.9% (72 of 814). Food insecurity and eating less declined more among people who received UI but not to the level of those who remained employed (eFigure 2 in the Supplement). Participants in the sample who were Hispanic individuals; were Black, American Indian or Alaska Native, Asian, or mixed race individuals; were younger; had lower income; were lesbian, gay, or bisexual individuals; were transgender or nonbinary individuals; and were single adults living with children were more likely to report food insecurity and eating less than other groups (Table 2).

**Table 2.** Ever Reporting Food Insecurity or Eating Less by Participant Characteristics

Characteristic	No./total No. (%)^a^	
	Food insecurity	Eating less
Total	415/1119 (37.1)	437/1119 (39.1)
Race and ethnicity		
Non-Hispanic		
White	158/588 (26.9)	186/588 (31.6)
Black	57/135 (42.2)	59/135 (43.7)
American Indian or Alaska Native	9/13 (69.2)	6/13 (46.2)
Asian	25/62 (40.3)	29/62 (46.8)
Hawaiian or Pacific Islander	0^b^	0^b^
Mixed race	27/54 (50.0)	25/54 (46.3)
Hispanic	139/265 (52.5)	132/265 (49.8)
Sex		
Women	281/732 (38.4)	291/732 (39.8)
Men	134/386 (34.7)	146/386 (37.7)
Income group, $		
<20 000	164/282 (58.2)	157/282 (55.7)
20 000-29 999	71/176 (40.3)	78/176 (44.3)
30 000-39 999	59/186 (31.7)	61/186 (32.8)
40 000-59 999	83/293 (28.3)	98/293 (33.4)
60 000-74 999	38/182 (20.9)	43/182 (23.6)
Age group, y		
18-29	109/223 (48.9)	114/223 (51.1)
30-39	116/251 (46.2)	122/251 (48.6)
40-49	89/187 (47.6)	97/187 (51.9)
50-59	63/229 (27.5)	66/229 (28.8)
≥60	38/229 (16.6)	38/229 (16.6)
Sexual orientation		
LGB	63/126 (50.0)	72/126 (57.1)
Heterosexual	352/993 (35.4)	365/993 (36.8)
Gender identity		
Cisgender	405/1100 (36.8)	426/1100 (38.7)
Transgender or nonbinary	10/19 (52.6)	11/19 (57.9)
Adult only households, no children	249/759 (32.8)	273/759 (36.0)
Single	104/268 (38.8)	108/268 (40.3)
≥2	148/497 (29.8)	168/497 (33.8)
Households with children	166/360 (46.1)	164/360 (45.6)
1 adult	28/47 (59.6)	26/47 (55.3)
≥2 adults	135/307 (44.0)	135/307 (44.0)

Abbreviation: LGB, lesbian, gay, or bisexual.

^a^ Reflects the percent of participants who ever reported food insecurity or eating less due to a lack of money or resources by participant characteristic. Percentages are unweighted.

^b^ Sample was too small for analysis.

### Difference-in-Differences Estimates

In our primary analysis, we found that UI receipt was associated with a 4.3 (95% CI, 1.8-6.9) percentage point decrease in food insecurity in our sample of people who had lost work during the COVID-19 epidemic (Table 3). This is equivalent to a 35.0% relative reduction in food insecurity from the average of 12.3% during the full study period. UI was also associated with a 5.7 (95% CI, 3.0-8.4) percentage point decrease in eating less. This is equivalent to a 47.9% relative reduction in eating less, from the mean of 11.9% during the full study period. Current employment was associated with a 4.1 (95% CI, 2.5-5.9) percentage point decrease in food insecurity and a 5.0 (95% CI, 2.3-6.0) percentage point decrease in eating less. The federal stimulus payment and SNAP were not associated with a change in food insecurity (federal stimulus payment: −1.3 [95% CI, −4.2 to 1.5] percentage points; SNAP: −0.5 [95% CI, −3.7 to 2.8] percentage points) or eating less (federal stimulus payment: −1.0 [ 95% CI, −4.1 to 2.0] percentage points; SNAP: −1.1 [95% CI: −4.7 to 2.5] percentage points).

**Table 3.** Main Difference-in-Differences Estimates of the Association Between Unemployment Insurance and Outcomes of Food Insecurity and Eating Less

Variable	Change, percentage points (95% CI)^a^					
	Unemployment insurance		Unemployment insurance and CARES Act supplement		Unemployment insurance amount	
	Food insecurity	Eating less	Food insecurity	Eating less	Food insecurity	Eating less
Unemployment insurance						
Only unemployment insurance	−4.32 (−6.87 to −1.76)	−5.69 (−8.43 to −2.96)	NA	NA	NA	NA
With CARES $600/wk supplement	NA	NA	−5.85 (−8.43 to −3.27)	−6.77 (−9.50 to −4.04)	NA	NA
Without CARES $600/wk supplement	NA	NA	−3.00 (−5.94 to −0.06)	−4.65 (−7.72 to −1.58)	NA	NA
Unemployment insurance amount, $						
0	NA	NA	NA	NA	[Reference]	[Reference]
1-300	NA	NA	NA	NA	−3.29 (−7.24 to −0.66)	−4.50 (−8.84 to −0.15)
301-600	NA	NA	NA	NA	−2.61 (−6.27 to 1.05)	−6.20 (−10.51 to −1.89)
601-900	NA	NA	NA	NA	−3.75 (−7.02 to −0.49)	−5.76 (−9.54 to −1.98)
901-1200	NA	NA	NA	NA	−7.25 (−11.15 to −3.35)	−4.82 (−8.60 to −1.04)
1201-1500	NA	NA	NA	NA	−7.02 (−10.93 to −3.10)	−6.46 (−10.01 to −2.91)
≥1500	NA	NA	NA	NA	−7.49 (−10.58 to −4.41)	−8.91 (−12.54 to −5.29)
Stimulus payment	−1.34 (−4.17 to 1.48)	−1.01 (−4.06 to 2.04)	−1.32 (−4.15 to 1.50)	−1.00 (−4.05 to 2.05)	−0.96 (−3.71 to 1.78)	−0.56 −(3.59 to 2.48)
SNAP	−0.45 (−3.70 to 2.81)	−1.09 (−4.72 to 2.54)	−0.46 (−3.72 to 2.79)	−1.11 (−4.73 to 2.51)	−0.39(−3.82 to 3.05)	−1.03 (−4.78 to 2.72)
Currently employed	−4.13 (−5.85 to −2.40)	−5.01 (−6.86 to −3.16)	−4.07 (−5.76 to −2.37)	−4.92 (−6.74 to −3.11)	−4.07 (−5.81 to −2.33)	−5.03 (−6.89 to −3.17)
Study wave						
Apr 1-28	[Reference]	[Reference]	[Reference]	[Reference]	[Reference]	[Reference]
Apr 15-May 12	−3.75 (−6.85 to −0.65)	−5.41 (−8.67 to −2.16)	−3.63 (−6.73 to −0.54)	−5.33 (−8.59 to −2.08)	−3.72 (−6.83 to −0.61)	−5.47 (−8.75 to −2.19)
Apr 29-May 26	−6.43 (−9.81 to −3.04)	−7.63 (−11.26 to −4.01)	−6.22 (−9.59 to −2.84)	−7.48 (−11.10 to −3.86)	−6.44 (−9.78 to −3.10)	−7.62 (−11.27 to −3.98)
May 13-Jun 9	−8.98 (−12.57 to −5.38)	−9.60 (−13.49 to −5.71)	−8.72 (−12.30 to −5.14)	−9.42 (−13.30 to −5.53)	−9.09 (−12.59 to −5.58)	−9.68 (−13.58 to −5.79)
May 27-Jun 23	−8.30 (−11.90 to −4.70)	−10.69 (−14.68 to −6.71)	−8.06 (−11.64 to −4.47)	−10.52 (−14.50 to −6.53)	−8.24 (−11.80 to −4.67)	−10.96 (−14.94 to −6.99)
Jun 10-Jul 8	−9.07 (−12.76 to −5.38)	−10.66 (−14.60 to −6.71)	−8.78 (−12.46 to −5.10)	−10.43 (−14.38 to −6.48)	−9.21 (−12.85 to −5.58)	−10.93 (−14.88 to −6.98)
Jun 24-Jul 22	−8.45 (−12.15 to −4.74)	−11.33 (−15.33 to −7.34)	−8.15 (−11.84 to −4.45)	−11.11 (−15.11 to −7.12)	−8.29 (−11.96 to −4.63)	−11.34 (−15.35 to −7.33)
Jul 8-Aug 5	−8.38 (−12.11 to −4.65)	−10.11 (−14.17 to −6.05)	−8.06 (−11.79 to −4.33)	−9.86 (−13.93 to −5.79)	−8.26 (−11.97 to −4.55)	−10.45 (−14.53 to −6.38)
Jul 22-Aug 19	−8.21 (−11.96 to −4.46)	−9.53 (−13.59 to −5.47)	−7.91 (−11.66 to −4.17)	−9.30 (−13.36 to −5.24)	−8.45 (−12.24 to −4.67)	−9.56 (−13.64 to −5.48)
Aug 5-Sep 2	−7.82 (−11.60 to −4.05)	−9.98 (−14.16 to −5.80)	−7.83 (−11.62 to −4.04)	−9.97 (−14.16 to −5.79)	−8.56 (−12.36 to −4.76)	−10.35 (−14.59 to −6.11)
Aug 19-Sep 16	−9.43 (−13.26 to −5.60)	−10.71 (−14.84 to −6.59)	−9.73 (−13.59 to −5.86)	−10.95 (−15.10 to −6.79)	−9.82 (−13.65 to −5.99)	−10.89 (−15.04 to −6.73)
Sep 2-Sep 30	−9.30 (−13.09 to −5.51)	−10.03 (−14.23 to −5.84)	−9.60 (−13.43 to −5.77)	−10.27 (−14.49 to −6.05)	−9.77 (−13.56 to −5.99)	−10.78 (−15.04 to −6.53)
Sep 16-Oct 14	−10.46 (−14.22 to −6.70)	−11.14 (−15.32 to −6.97)	−10.76 (−14.56 to −6.96)	−11.38 (−15.58 to −7.18)	−10.92 (−14.70 to −7.14)	−11.57 (−15.79 to −7.36)
Sep 30-Oct 27	−11.27 (−15.01 to −7.52)	−10.82 (−14.96 to −6.68)	−11.55 (−15.33 to −7.76)	−11.04 (−15.21 to −6.87)	−11.69 (−15.45 to −7.93)	−11.39 (−15.57 to −7.21)
Oct 14-Nov 11	−9.80 (−13.62 to −5.99)	−11.49 (−15.53 to −7.45)	−10.09 (−13.95 to −6.23)	−11.72 (−15.79 to −7.65)	−10.22 (−14.09 to −6.35)	−11.83 (−15.93 to −7.73)
Constant	24.16 (21.55 to 26.77)	25.58 (22.88 to 28.28)	24.07 (21.47 to 26.68)	25.5 (22.80 to 28.19)	23.82 (21.23 to 26.40)	25.34 (22.65 to 28.04)

Abbreviations: CARES, Coronavirus Aid, Relief, and Economic Security; NA, not applicable; SNAP, Supplemental Nutrition Assistance Program.

^a^ Estimates are unweighted. For unemployment insurance and unemployment insurance and CARES, there were 1119 participants, with 12 596 observations. For unemployment amount, there were 1118 participants with 12 004 observations because fewer participants reported unemployment insurance amount.

**Figure 1.**
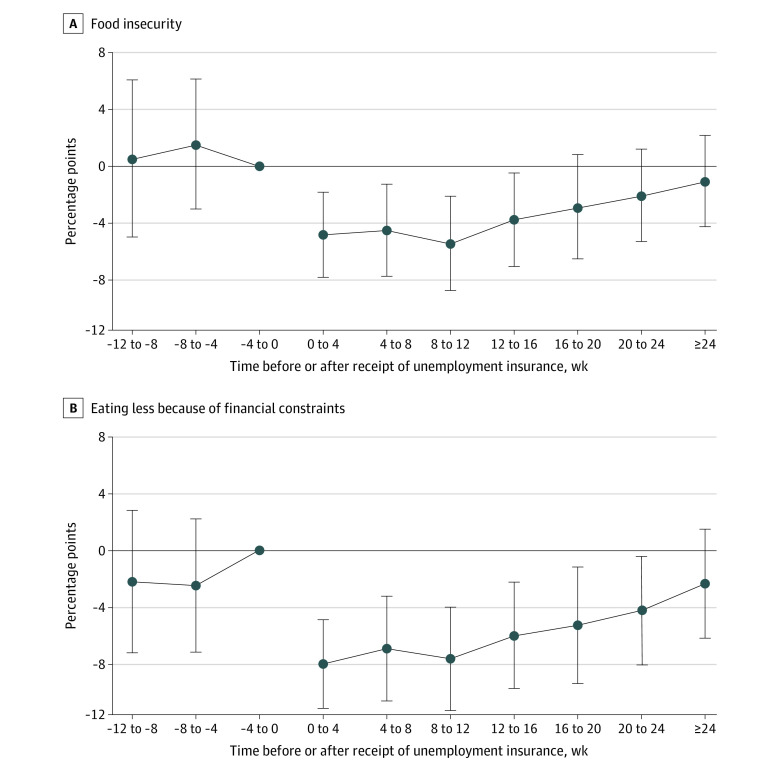
Changes in Food Insecurity and Eating Less Due to Financial Constraints Before and After Unemployment Insurance Receipt, Event Study Estimates Estimates of the association between unemployment insurance and food insecurity or eating less are based on running the main regression with all covariates and replacing the main unemployment insurance exposure variable with binary indicators for the period immediately prior to receipt of unemployment insurance among those who received unemployment insurance with individuals whose unemployment insurance status did not change during the study period set to 0. All estimates are adjusted for stimulus payments, receipt of Supplemental Nutrition Assistance Program benefits within the past 4 weeks, current employment status, and study wave as well as individual-level fixed effects. The reference period is the period immediately prior to receipt of unemployment insurance. Standard errors are clustered by individual. The error bars represent 95% CIs.

Results from event study analyses were similar (Figure 1). Reductions in food insecurity and eating less were greatest in the periods immediately following initial receipt of UI. Food insecurity decreased 4.9 (95% CI, 1.8 to 8.0) percentage points, and eating less due to financial constraints decreased 8.0 (95% CI, 4.9 to 11.1) percentage points 0 to 4 weeks after UI receipt. The event study estimates do not suggest differential trends in food insecurity or eating less prior to receipt of UI benefits.

**Figure 2.**
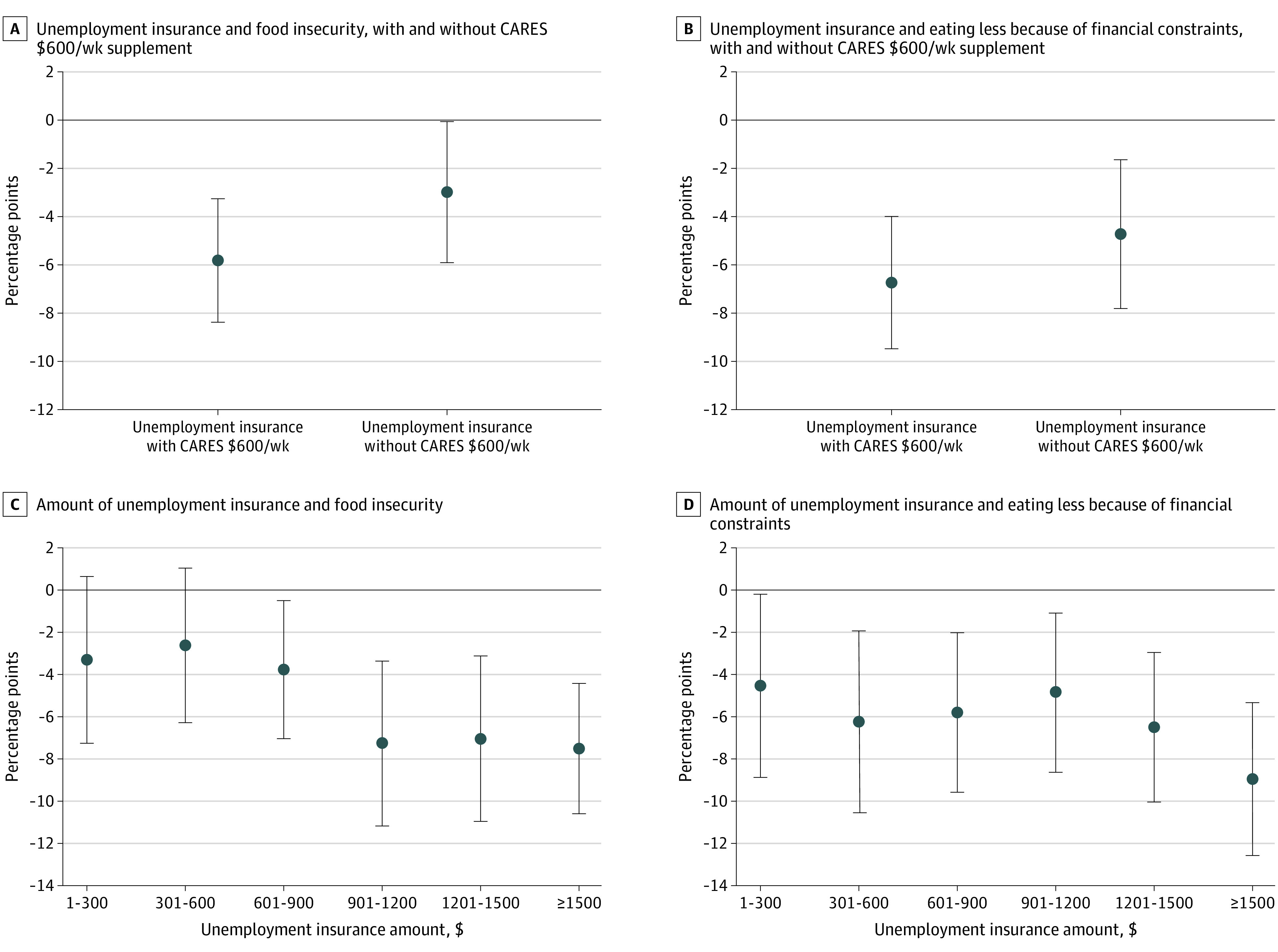
Estimates of the Association of $600/wk Supplement Unemployment and the Amount of Unemployment Insurance With Food Insecurity and Eating Less Receiving unemployment insurance with the $600/wk supplement was associated with a significantly greater reduction in food insecurity (*P* = .02), while there was not a significant difference in the association between unemployment insurance with and without the $600/wk supplement and eating less (*P* = .07). All estimates are adjusted for stimulus payments, receipt of Supplemental Nutrition Assistance Program benefits within the past 4 weeks, current employment status, and study wave as well as individual-level fixed effects. Standard errors are clustered by individual. The error bars represent 95% CIs.

In the secondary analyses, UI with the CARES Act $600/wk UI supplement was associated with a greater reduction in food insecurity (−5.9 [95% CI, −8.4 to −3.3] percentage points) than unemployment insurance without the supplement (−3.0 [95% CI, −5.9 to −0.1] percentage points; *P* = .02). Unemployment insurance with and without the supplement were both associated with reductions in eating less; there was no difference between the 2 estimates (with supplement: −6.8 [95% CI, −9.5 to −4.0] percentage points; without supplement: −4.7 [95% CI, −7.7 to −1.6] percentage points; *P* = .07) (Table 3, Figure 2A, and Figure 2B). Larger UI amounts were associated with greater reductions in food insecurity and eating less (Table 3, Figure 2C, and Figure 2D).

### Sensitivity Analyses

Sensitivity analyses were consistent with the main result for individuals who participated in the UCA survey in each month (eTable 3 in the Supplement), for models with survey weights (eTable 4 in the Supplement), for logistic rather than linear models (eTable 5 in the Supplement), and when we restricted the analysis to those currently unemployed (eTable 6 in the Supplement). Estimated associations were larger for participants living in households with incomes less than $20 000 than for the full sample living in households with income of less than $75 000 (eTable 7 in the Supplement). Estimates remained consistent when we updated data from July to November.

## Discussion

In this national cohort study, we found that 37% of individuals living in households earning less than $75 000 who lost work during the COVID-19 pandemic reported food insecurity between April and November 2020, and 39% reported eating less due to financial constraints. Food insecurity was concentrated among those living in households with less than $20 000 in earnings, among whom 58% reported food insecurity and 56% reported eating less. In comparison, 11% of all households and 35% of households below the federal poverty limit reported any food insecurity in 2019.^23^ Structural inequities have shaped large racial and ethnic disparities in unemployment and in food insecurity as well as disparities by sexual orientation and gender identity.

Receipt of UI was associated with a 35% reduction in food insecurity and a 48% decline in eating less due to financial constraints among people with household earnings of less than $75 000 who lost their jobs during the COVID-19 pandemic. Larger amounts of UI were associated with greater reductions in food insecurity. Reductions in food insecurity were greatest immediately after receipt of UI and declined over subsequent periods, possibly due to the CARES $600/wk federal supplement ending in July. The CARES supplement was associated with further reductions in food insecurity.

Our findings are consistent with recent work showing that household spending increased immediately following receipt of UI for households that began receiving UI in April 2020.^24^ Our findings are also consistent with historical^13^ and recent^14^ evidence that UI is associated with reduced food insecurity. While there has been some concern that supplemental UI deterred people from seeking work, evidence suggests that it did not reduce employment.^25^

UI and federal UI supplementation play an important role in ensuring access to basic needs, such as food and housing, in times of crisis. Food banks may not be accessible or sustainably financed^26,27^ and impose significant costs on users, such as wait times and limited selection. Cash UI benefits may also be easier for recipients to use more efficiently than restricted funds, such as SNAP benefits.^28^ The effectiveness of UI in reducing food insecurity going forward is likely to depend on the continued generosity of federal policies, such as the CARES $600/wk UI supplement.

Food insecurity is a persistent challenge in the United States.^12^ Policies expanding UI eligibility, amount, and duration to levels more consistent with other high-income countries^32^ may be an effective approach to reducing enduring food insecurity.

Our study was not well powered to evaluate the association between receipt of SNAP benefits and food insecurity or eating less. There were not broad changes to SNAP eligibility by November 2020, except for families previously receiving free or reduced price meals for school-aged children^29^; therefore, the associations of SNAP would mostly be subsumed by individual fixed effects in this study.

### Limitations

As with all difference-in-differences analyses, particularly those conducted in the rapidly changing policy context of COVID-19,^30^ our study has clear limitations. UI and stimulus payments were often delivered in close temporal proximity to each other, making it difficult to fully distinguish the effects of each, even after covariate adjustment. Measurements of both the outcome and exposure rely on self-report, which may be biased. Finally, the web-based sample may not be truly representative of the US population and individuals from underrepresented racial and ethnic groups. However, rates of unemployment^31^ and food insecurity^12^ in our sample were similar to estimates from other surveys.

## Conclusions

In this study, UI was associated with a 35% reduction in reporting any food insecurity and a 48% decline in eating less due to financial constraints. Larger amounts of UI were associated with larger reductions in food insecurity. Millions of people are expected to lose UI when the federal UI provisions expire on March 14, 2021. Policy makers may wish to consider continued expansion of UI eligibility, duration, and amount as an approach to reducing food insecurity during and after the COVID-19 pandemic.

## Notes

Supplement. **eAppendix. **Statistical Model **eTable 1. **UCA Survey Waves and Dates **eFigure 1. **Food Insecurity Over Time by Household Income Group **eTable 2. **Unemployment **eFigure 2. **Temporal Trends in Food Insecurity and Eating Less Among Those Who Did and Did Not Receive Unemployment Insurance **eTable 3. **Main Difference-in-Differences Estimates of the Association Between Unemployment Insurance and Outcomes of Food Insecurity and Eating Less Among Participants Who Participated in the UCA Survey at Least Once in Each Month, April to October **eTable 4. **Main Difference-in-Differences Estimates of the Association Between Unemployment Insurance and Outcomes of Food Insecurity and Eating Less With Survey Weights Included **eTable 5. **Main Difference-in-Differences Estimates of the Association Between Unemployment Insurance and Outcomes of Food Insecurity and Eating Less Based on Logistic Regression **eTable 6. **Main Difference-in-Differences Estimates of the Association Between Unemployment Insurance and Outcomes of Food Insecurity and Eating Less Among Those Currently Unemployed **eTable 7. **Main Difference-in-Differences Estimates of the Association Between Unemployment Insurance and Outcomes of Food Insecurity and Eating Less in Very Low-Income Households (<$20 000/Year)
