# Supplementary material for: Association Between Receipt of Unemployment Insurance and Food Insecurity Among People Who Lost Employment During the COVID-19 Pandemic in the United States
Source: JAMA Netw Open. 2021 Jan 29;4(1):e2035884. doi: 10.1001/jamanetworkopen.2020.35884 (PMC7846943; doi:10.1001/jamanetworkopen.2020.35884)
Supplement: Supplement. — eAppendix. Statistical Model eTable 1. UCA Survey Waves and Dates eFigure 1. Food Insecurity Over Time by Household Income Group eTable 2. Unemployment eFigure 2. Temporal Trends in Food Insecurity and Eating Less Among Those Who Did and Did Not Receive Unemployment Insurance eTable 3. Main Difference-in-Differences Estimates of the Association Between Unemployment Insurance and Outcomes of Food Insecurity and Eating Less Among Participants Who Participated in the UCA Survey at Least Once in Each Month, April to October eTable 4. Main Difference-in-Differences Estimates of the Association Between Unemployment Insurance and Outcomes of Food Insecurity and Eating Less With Survey Weights Included eTable 5. Main Difference-in-Differences Estimates of the Association Between Unemployment Insurance and Outcomes of Food Insecurity and Eating Less Based on Logistic Regression eTable 6. Main Difference-in-Differences Estimates of the Association Between Unemployment Insurance and Outcomes of Food Insecurity and Eating Less Among Those Currently Unemployed eTable 7. Main Difference-in-Differences Estimates of the Association Between Unemployment Insurance and Outcomes of Food Insecurity and Eating Less in Very Low-Income Households (<$20 000/Year) [file jamanetwopen-e2035884-s001.pdf]

Supplemental Online Content

Raifman J, Bor J, Venkataramani A. Association between receipt of unemployment insurance and food insecurity among people who lost employment during the COVID-19 pandemic in the United States. *JAMA Netw Open*. 2021;4(1):e2035884.
doi:10.1001/jamanetworkopen.2020.35884

eAppendix. Statistical Model
eTable 1. UCA Survey Waves and Dates
eFigure 1. Food Insecurity Over Time by Household Income Group
eTable 2. Unemployment
eFigure 2. Temporal Trends in Food Insecurity and Eating Less Among Those Who Did and Did Not Receive Unemployment Insurance
eTable 3. Main Difference-in-Differences Estimates of the Association Between Unemployment Insurance and Outcomes of Food Insecurity and Eating Less Among Participants Who Participated in the UCA Survey at Least Once in Each Month, April to October
eTable 4. Main Difference-in-Differences Estimates of the Association Between Unemployment Insurance and Outcomes of Food Insecurity and Eating Less With Survey Weights Included
eTable 5. Main Difference-in-Differences Estimates of the Association Between Unemployment Insurance and Outcomes of Food Insecurity and Eating Less Based on Logistic Regression
eTable 6. Main Difference-in-Differences Estimates of the Association Between Unemployment Insurance and Outcomes of Food Insecurity and Eating Less Among Those Currently Unemployed
eTable 7. Main Difference-in-Differences Estimates of the Association Between Unemployment Insurance and Outcomes of Food Insecurity and Eating Less in Very Low-Income Households (<$20 000/Year)

This supplemental material has been provided by the authors to give readers additional information about their work.

© 2021 Raifman J et al. *JAMA Network Open.*

eAppendix. Statistical Model

Our statistical model is presented in **Equation 1**, where FI_{it} is a binary indicator for food insecurity, UI_{it} is a binary indicator that switches from 0 to 1 if the individual begins receiving unemployment insurance, S_{it} is a binary indicator that switches from 0 to 1 if the individual receives the stimulus payment, $SNAP_{it}$ is a binary indicator for receiving SNAP benefits in the past month, I_i is individual fixed effects, and t_t is period fixed effects. In the event study, we replaced UI_{it} with dummy variables indicating the number of periods prior to or following receipt of unemployment in insurance.

$$FI_{it} = UI_{it} + S_{it} + SNAP_{it} + CurrentJob_{it} + I_i + t_t + \varepsilon_{ist} \qquad (1)$$

© 2021 Raifman J et al. *JAMA Network Open.*

eTable 1. UCA Survey Waves and Dates

UCA survey wave	Dates
Not included because different sampling strategy and questions	March 10 – March 31
1	April 1 – April 28
2	April 15 – May 12
3	April 29 – May 26
4	May 13 – June 9
5	May 27 – June 23
6	June 10 – July 8
7	June 24 – July 22
8	July 8 – August 5
9	July 22 – August 19
10	August 5 – September 2
11	August 19 – September 16
12	September 2 – September 30
13	September 16 – October 14
14	September 30 – October 27
15	October 14 – November 11

Note: New UCA surveys are fielded every two weeks. Each day one fourteenth of participants are invited to take the survey, and participants have two weeks to take the survey – meaning that the total field period for each wave of the survey is 4 weeks and there is overlap between waves. Participants are incentivized to respond to the survey on the day they are invited to participate.

eFigure 1. Food Insecurity Over Time by Household Income Group

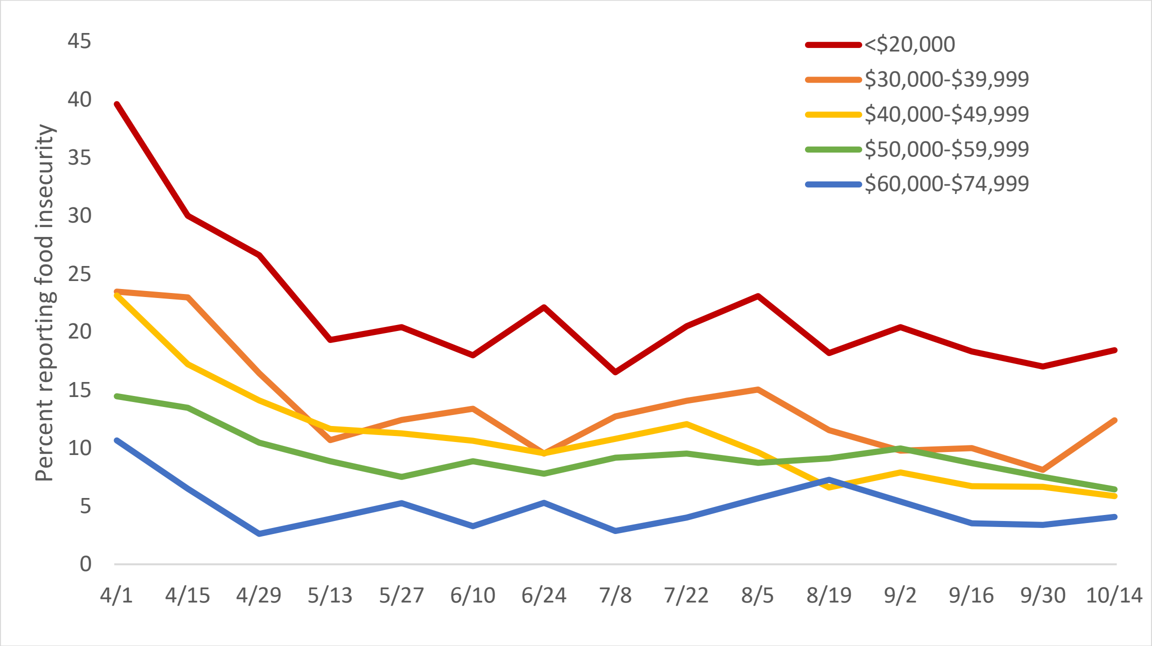

Notes: We conducted the main analysis among people in households earning less than $75,000 because more than 20% of people in all income groups below $75,000 reported food insecurity at some point during the COVID-19 pandemic. We conducted a subgroup analysis among people in households earning less than $20,000 because they were most likely to report food insecurity, with 58% reporting food insecurity at some point during the pandemic.

© 2021 Raifman J et al. *JAMA Network Open.*

eTable 2. Unemployment

Characteristic	(1)<br>N with household income <$75,000	(2)<br>N unemployed	(3)<br>% unemployed at any point
**Total**	2,319	1,119	48.3
**Race and ethnicity**			
Non-Hispanic White	1,327	588	44.3
Non-Hispanic Black	243	135	55.6
Non-Hispanic American Indian or Alaska Native	30	13	43.3
Non-Hispanic Asian	125	62	49.6
Non-Hispanic Hawaiian/Pacific Islander	8	2	25.0
Non- Hispanic Mixed Race	97	54	55.7
Hispanic	488	265	54.3
Not reported	1	0	0.0
**Sex**			
Female	1466	732	49.9
Male	853	387	45.4
**Income group**			
<$20,000	368	282	76.6
$20,000 to $29,999	306	176	57.5
$30,000 to $39,999	388	186	47.9
$40,000 to $59,999	743	293	39.4
$60,000 to $74,999	514	182	35.4
**Age group**			
18 to 29 years	399	223	55.9
30 to 39 years	577	251	43.5
40 to 49 years	459	187	40.7
50 to 59 years	470	229	48.7
≥60 years	414	229	55.3
**Sexual orientation**			
LGB	222	126	56.8
Heterosexual	2097	993	47.4
**Gender identity**			
Cisgender	2,293	1,100	48.0
Transgender or non-binary	26	19	73.1
**Adult households**	1559	765	49.1
Single adult, no children	532	268	50.4
Two adults, no children	1027	497	48.4
**Households with children**	760	354	46.6
1 adult, with children	96	47	49.0
≥2 adults, with children	664	307	46.2

© 2021 Raifman J et al. *JAMA Network Open.*

Note: We present the number and percentage of people with each characteristic who lost employment. Characteristics are based on the first observation for each participant in the sample. All observations are from individuals who participated in at least 2 waves of the UCA survey. Numbers and percentages represent the full sample and are unweighted.

© 2021 Raifman J et al. *JAMA Network Open.*

eFigure 2. Temporal Trends in Food Insecurity and Eating Less Among Those Who Did and Did Not Receive Unemployment Insurance

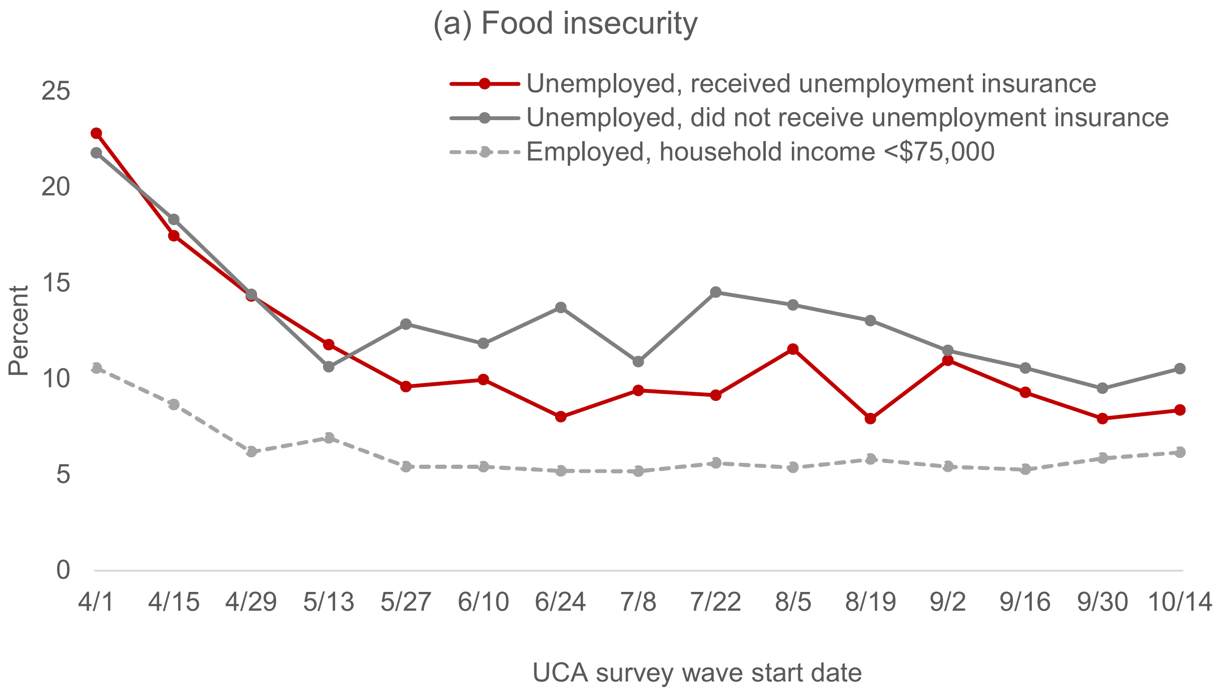

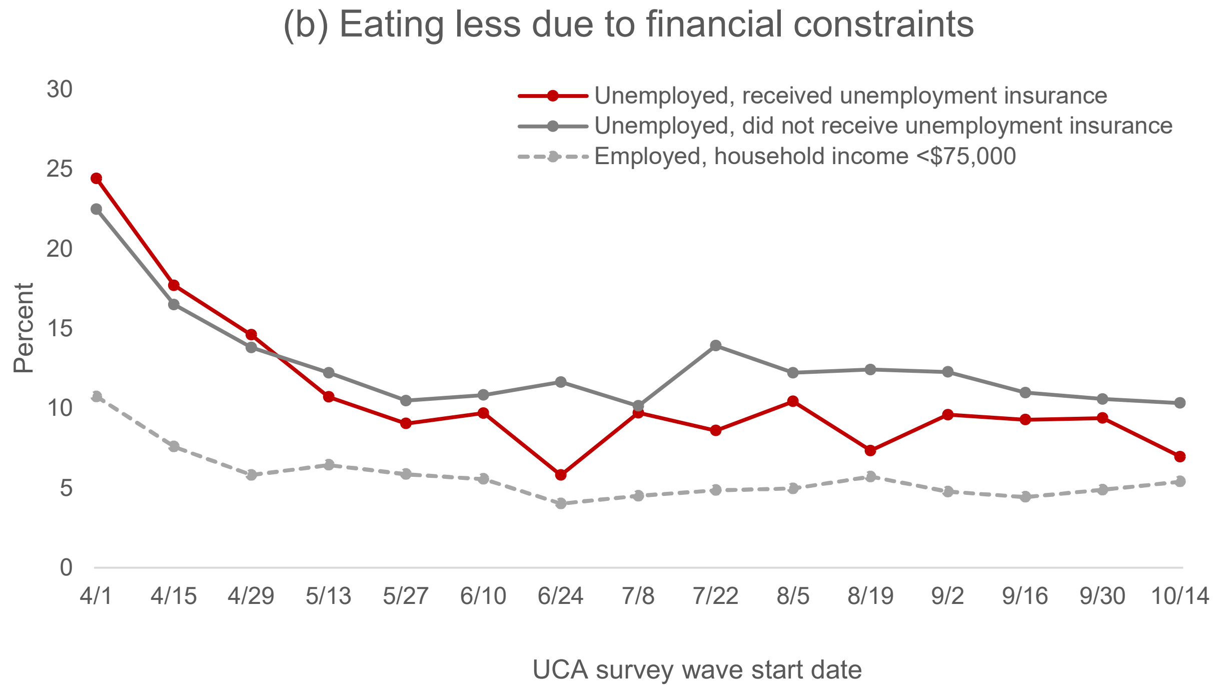

© 2021 Raifman J et al. *JAMA Network Open.*

eTable 3. Main Difference-in-Differences Estimates of the Association Between Unemployment Insurance and Outcomes of Food Insecurity and Eating Less Among Participants Who Participated in the UCA Survey at Least Once in Each Month, April to October

	**(1) Unemployment insurance**			**(2) Unemployment insurance and CARES**			**(3) Unemployment insurance amount**	
	Participants: 678 Observations: 9492			Participants: 678 Observations: 9492			Participants: 678 Observations: 9041	
	**Percentage points**	**95% CI**		**Percentage points**	**95% CI**		**Percentage points**	**95% CI**
**(a) Food insecurity**								
**Unemployment insurance**	-4.07	-7.13 - -1.00						
**Unemployment insurance with CARES $600/week supplement**				-5.13	-8.06 - -2.21			
**Unemployment insurance without CARES $600/week supplement**				-3.05	-6.64 - 0.54			
**Unemployment insurance amount**								
No unemployment insurance							Reference	
$1 to 300							-3.45	-8.28 - 1.38
$301 to 600							-3.46	-7.59 - 0.66
$601 to 900							-3.64	-7.39 - 0.11
$901 to 1200							-5.57	-10.13 - -1.01
$1201 to 1500							-4.49	-8.63 - -0.35
≥$1500							-6.30	-9.15 - -3.46
**Stimulus payment**	-0.97	-4.12 - 2.18		-0.97	-4.13 - 2.18		-0.50	-3.5

© 2021 Raifman J et al. *JAMA Network Open.*

								2 - 2.51
**SNAP**	-1.14	-4.72 - 2.43		-1.17	-4.74 - 2.40		-0.91	-4.68 - 2.85
**Currently employed**	-4.34	-6.22 - -2.46		-4.27	-6.12 - -2.42		-4.30	-6.18 - -2.41
**Study wave**								
April 1 – April 28	Reference			Reference			Reference	
April 15 – May 12	-6.27	-10.03 - -2.51		-5.04	-8.57 - -1.52		-6.48	-10.26 - -2.69
April 29 – May 26	-8.48	-12.66 - -4.29		-7.01	-10.83 - -3.18		-8.69	-12.89 - -4.49
May 13 – June 9	-10.43	-14.97 - -5.88		-8.69	-12.80 - -4.58		-10.70	-15.23 - -6.17
May 27 – June 23	-10.58	-15.25 - -5.92		-7.89	-12.04 - -3.74		-11.26	-15.89 - -6.62
June 10 – July 8	-11.20	-15.78 - -6.61		-9.15	-13.34 - -4.97		-11.68	-16.25 - -7.10
June 24 – July 22	-12.46	-17.09 - -7.83		-8.70	-12.95 - -4.46		-12.68	-17.30 - -8.06
July 8 – Aug 5	-11.15	-15.88 - -6.42		-8.24	-12.50 - -3.98		-11.77	-16.49 - -7.04
July 22 – Aug 19	-10.70	-15.48 - -5.92		-7.67	-12.00 - -3.33		-11.09	-15.

© 2021 Raifman J et al. *JAMA Network Open.*

								85 - - 6.32
Aug 5 – Sept 2	-10.41	-15.23 - -5.60		-7.83	-12.09 - - 3.56		-11.11	-15.97 - - 6.24
Aug 19 – Sept 16	-11.46	-16.26 - -6.66		-9.46	-13.87 - - 5.04		-11.97	-16.78 - - 7.16
Sept 2 – Sept 30	-10.67	-15.52 - -5.83		-9.26	-13.68 - - 4.84		-11.82	-16.73 - - 6.90
Sept 16 – Oct 14	-11.01	-15.83 - -6.18		-10.53	-14.82 - - 6.23		-11.82	-16.70 - - 6.95
Sept 30 – Oct 27	-11.04	-15.84 - -6.25		-10.75	-15.06 - - 6.43		-11.84	-16.68 - - 6.99
Oct 14 – Nov 11	-12.06	-16.73 - -7.39		-10.22	-14.56 - - 5.88		-12.67	-17.40 - - 7.94
**Constant**	20.90	18.00 - 23.80		20.83	17.93 - 23.73		20.49	17.62 - 23.36
**(b) Eating less due to financial constraints**								
**Unemployment insurance**	-5.54	-8.82 - -2.27						
**Unemployment insurance with CARES $600/week supplement**				-6.83	-10.06 - - 3.60			
**Unemployment insurance without CARES $600/week supplement**				-4.19	-7.86 - -0.51			
**Unemployment insurance amount**								
No unemployment insurance							Reference	
$1 to 300							-3.68	-8.95 -

© 2021 Raifman J et al. *JAMA Network Open.*

								1.60
$301 to 600							-6.18	-10.95 - -1.41
$601 to 900							-5.12	-9.87 - -0.38
$901 to 1200							-5.85	-10.19 - -1.51
$1201 to 1500							-6.14	-10.42 - -1.87
≥$1500							-8.67	-13.13 - -4.21
**Stimulus payment**	-0.91	-4.46 - 2.65		-0.91	-4.47 - 2.65		-0.20	-3.72 - 3.32
**SNAP**	-1.84	-6.34 - 2.66		-1.87	-6.36 - 2.62		-1.40	-6.02 - 3.21
**Currently employed**	-5.04	-6.97 - -3.12		-4.93	-6.82 - -3.05		-5.09	-7.02 - -3.16
**Study wave**								
April 1 – April 28	Reference			Reference			Reference	
April 15 – May 12	-6.27	-10.03 - -2.51		-6.18	-9.93 - -2.42		-6.48	-10.26 - -2.69
April 29 – May 26	-8.48	-12.66 - -4.29		-8.30	-12.48 - -4.13		-8.69	-12.89 - -

								4.49
May 13 – June 9	-10.43	-14.97 - -5.88		-10.21	-14.74 - -5.68		-10.70	-15.23 - -6.17
May 27 – June 23	-10.58	-15.25 - -5.92		-10.37	-15.04 - -5.70		-11.26	-15.89 - -6.62
June 10 – July 8	-11.20	-15.78 - -6.61		-10.97	-15.55 - -6.38		-11.68	-16.25 - -7.10
June 24 – July 22	-12.46	-17.09 - -7.83		-12.19	-16.83 - -7.56		-12.68	-17.30 - -8.06
July 8 – Aug 5	-11.15	-15.88 - -6.42		-10.88	-15.60 - -6.15		-11.77	-16.49 - -7.04
July 22 – Aug 19	-10.70	-15.48 - -5.92		-10.45	-15.21 - -5.68		-11.09	-15.85 - -6.32
Aug 5 – Sept 2	-10.41	-15.23 - -5.60		-10.41	-15.24 - -5.58		-11.11	-15.97 - -6.24
Aug 19 – Sept 16	-11.46	-16.26 - -6.66		-11.75	-16.57 - -6.92		-11.97	-16.78 - -7.16
Sept 2 – Sept 30	-10.67	-15.52 - -5.83		-10.95	-15.83 - -6.08		-11.82	-16.73 - -6.90
Sept 16 – Oct 14	-11.01	-15.83 - -6.18		-11.29	-16.15 - -6.43		-11.82	-16.70 - -6.95

© 2021 Raifman J et al. *JAMA Network Open.*

Sept 30 – Oct 27	-11.04	-15.84 - -6.25		-11.30	-16.14 - - 6.47		-11.84	-16.68 - - 6.99
Oct 14 – Nov 11	-12.06	-16.73 - -7.39		-12.34	-17.05 - - 7.63		-12.67	-17.40 - - 7.94
**Constant**	23.14	20.09 - 26.19		23.04	20.00 - 26.08		22.88	19.85 - 25.90

© 2021 Raifman J et al. *JAMA Network Open.*

eTable 4. Difference-in-Differences Estimates of the Association Between Unemployment Insurance and Outcomes of Food Insecurity and Eating Less With Survey Weights Included

	(1) Unemployment insurance			(2) Unemployment insurance and CARES			(3) Unemployment insurance amount	
	Participants: 1119 Observations: 12,596			Participants: 1119 Observations: 12,596			Participants: 1118 Observations: 12,004	
	**Percentage points**	**95% CI**		**Percentage points**	**95% CI**		**Percentage points**	**95% CI**
**(a) Food insecurity**								
**Unemployment insurance**	-3.50	-6.40 - -0.59						
**Unemployment insurance with CARES $600/week supplement**				-5.63	-8.54 - -2.73			
**Unemployment insurance without CARES $600/week supplement**				-1.89	-5.19 - 1.42			
**Unemployment insurance amount**								
No unemployment insurance							Reference	
$1 to 300							-3.19	-7.40 - 1.02
$301 to 600							0.31	-4.23 - 4.86
$601 to 900							-2.50	-5.89 - 0.88
$901 to 1200							-8.63	-13.82 - -3.44
$1201 to 1500							-5.78	-9.11 - -2.45
≥$1500							-5.08	-8.65 - -1.51
**Stimulus payment**	-3.19	-6.62 - 0.23		-3.15	-6.57 - 0.28		-3.01	-6.38 -

© 2021 Raifman J et al. *JAMA Network Open.*

								0.36
**SNAP**	0.84	-3.33 - 5.00			-3.32 - 4.99		1.35	-3.02 - 5.72
**Currently employed**	-5.95	-8.02 - -3.88		-5.88	-7.93 - -3.83		-5.87	-7.91 - -3.82
**Study wave**								
April 1 – April 28	Reference			Reference			Reference	
April 15 – May 12	-4.06	-8.37 - 0.26		-3.95	-8.27 - 0.38		-4.28	-8.51 - -0.06
April 29 – May 26	-6.65	-11.18 - -2.12		-6.48	-11.02 - -1.94		-6.80	-11.24 - -2.35
May 13 – June 9	-6.07	-10.97 - -1.17		-5.82	-10.75 - -0.90		-6.41	-11.25 - -1.56
May 27 – June 23	-6.16	-10.97 - -1.34		-5.94	-10.77 - -1.11		-6.22	-11.03 - -1.41
	-7.45	-12.23 - -2.68		-7.23	-12.03 - -2.44		-7.79	-12.55 - -3.04
June 24 – July 22	-6.42	-11.27 - -1.56		-6.18	-11.05 - -1.30		-5.84	-10.66 - -1.01
July 8 – Aug 5	-6.76	-11.56 - -1.96		-6.47	-11.29 - -1.65		-6.49	-11.27 - -1.71
July 22 – Aug 19	-5.77	-10.64 - -0.90		-5.56	-10.45 - -0.68		-5.57	-10.38

								- -0.75
Aug 5 – Sept 2	-6.76	-11.62 - -1.91		-6.86	-11.72 - -1.99		-7.28	-12.07 - -2.49
Aug 19 – Sept 16	-7.49	-12.36 - -2.61		-7.90	-12.80 - -3.01		-8.01	-12.85 - -3.17
Sept 2 – Sept 30	-8.05	-12.88 - -3.22		-8.44	-13.28 - -3.61		-8.35	-13.12 - -3.58
Sept 16 – Oct 14	-9.64	-14.54 - -4.74		-10.03	-14.94 - -5.11		-10.23	-15.11 - -5.35
Sept 30 – Oct 27	-11.66	-16.59 - -6.74		-12.02	-16.96 - -7.09		-11.27	-16.17 - -6.37
Oct 14 – Nov 11	-8.89	-13.86 - -3.92		-9.26	-14.25 - -4.28		-8.66	-13.62 - -3.69
**Constant**	14.72	11.04 - 18.39		14.64	10.96 - 18.32		14.58	10.89 - 18.26
**(b) Eating less due to financial constraints**								
**Unemployment insurance**	-5.82	-8.85 - -2.79						
**Unemployment insurance with CARES $600/week supplement**				-6.84	-9.96 - -3.71			
**Unemployment insurance without CARES $600/week supplement**				-4.71	-8.00 - -1.41			
**Unemployment insurance amount**								
No unemployment insurance							Reference	
$1 to 300							-4.95	-9.55 - -0.36

© 2021 Raifman J et al. *JAMA Network Open.*

$301 to 600							-4.46	-9.55 - 0.62
$601 to 900							-5.90	-9.79 - -2.02
$901 to 1200							-2.89	-6.93 - 1.14
$1201 to 1500							-6.26	-9.68 - -2.85
≥$1500							-7.64	-11.71 - -3.58
**Stimulus payment**	-0.92	-4.73 - 2.88		-0.91	-4.72 - 2.90		-0.73	-4.54 - 3.08
**SNAP**	-0.41	-4.73 - 3.91		-0.43	-4.75 - 3.88		-0.24	-4.82 - 4.34
**Currently employed**	-7.10	-9.24 - -4.95		-6.96	-9.08 - -4.84		-6.94	-9.09 - -4.78
**Study wave**								
April 1 – April 28	Reference			Reference			Reference	
April 15 – May 12	-5.67	-10.24 - -1.10		-5.61	-10.19 - -1.04		-5.81	-10.31 - -1.30
April 29 – May 26	-7.41	-12.37 - -2.45		-7.33	-12.30 - -2.36		-7.59	-12.52 - -2.65
May 13 – June 9	-8.36	-13.87 - -2.86		-8.25	-13.76 - -2.74		-8.75	-14.

								29 - - 3.20
May 27 – June 23	-10.17	-15.33 - -5.01		-10.08	-15.25 - - 4.90		-10.53	-15.68 - - 5.38
June 10 – July 8	-9.62	-14.78 - -4.45		-9.49	-14.66 - - 4.31		-9.81	-14.98 - - 4.64
June 24 – July 22	-10.20	-15.46 - -4.94		-10.07	-15.35 - - 4.79		-10.21	-15.49 - - 4.93
July 8 – Aug 5	-9.47	-14.80 - -4.13		-9.31	-14.66 - - 3.96		-9.64	-14.99 - - 4.29
July 22 – Aug 19	-7.71	-12.95 - -2.46		-7.58	-12.84 - - 2.33		-7.49	-12.73 - - 2.25
Aug 5 – Sept 2	-10.51	-15.69 - -5.34		-10.56	-15.74 - - 5.37		-10.57	-15.76 - - 5.38
Aug 19 – Sept 16	-10.00	-15.24 - -4.76		-10.28	-15.53 - - 5.02		-10.32	-15.55 - - 5.08
Sept 2 – Sept 30	-8.53	-13.76 - -3.30		-8.80	-14.04 - - 3.56		-9.51	-14.73 - - 4.30
Sept 16 – Oct 14	-10.66	-15.93 - -5.39		-10.91	-16.19 - - 5.63		-10.84	-16.08 - - 5.60
Sept 30 – Oct 27	-11.69	-17.01 - -6.38		-11.93	-17.26 - - 6.61		-11.38	-16.68 - -

© 2021 Raifman J et al. *JAMA Network Open.*

								6.0 8
Oct 14 – Nov 11	-11.30	-16.51 - -6.09		-11.55	-16.77 - - 6.33		-11.18	- 16. 41 - - 5.9 5
**Constant**	15.72	11.82 - 19.61		15.61	11.72 - 19.50		15.57	11. 66 - 19. 48

© 2021 Raifman J et al. *JAMA Network Open.*

eTable 5. Main Difference-in-Differences Estimates of the Association Between Unemployment Insurance and Outcomes of Food Insecurity and Eating Less Based on logistic regression

	**(1) Unemployment insurance**			**(2) Unemployment insurance and CARES**			**(3) Unemployment insurance amount**	
	Participants: 1119 Observations: 12,596			Participants: 1119 Observations: 12,596			Participants: 1118 Observations: 12,004	
	**Percentage points**	**95% CI**		**Percentage points**	**95% CI**		**Percentage points**	**95% CI**
**(a) Food insecurity**								
**Unemployment insurance**	0.63	0.45 - 0.88						
**Unemployment insurance with CARES $600/week supplement**				0.43	0.28 - 0.66			
**Unemployment insurance without CARES $600/week supplement**				0.80	0.55 - 1.15			
**Unemployment insurance amount**								
No unemployment insurance							Reference	
$1 to 300							0.68	0.41 - 1.14
$301 to 600							0.88	0.45 - 1.73
$601 to 900							0.62	0.33 - 1.14
$901 to 1200							0.16	0.05 - 0.54
$1201 to 1500							0.22	0.06 - 0.82
≥$1500							0.23	0.10 - 0.56
**Stimulus payment**	0.69	0.46 - 1.03		0.69	0.46 - 1.03		0.69	0.45 - 1.06

© 2021 Raifman J et al. *JAMA Network Open.*

**SNAP**	1.04	0.72 - 1.50		1.04	0.72 - 1.50		1.08	0.73 - 1.60
**Currently employed**		0.34 - 0.59		0.45	0.35 - 0.59		0.44	0.34 - 0.58
**Study wave**								
April 1 – April 28	Reference			Reference			Reference	
April 15 – May 12	0.60	0.39 - 0.92		0.61	0.40 - 0.95		0.65	0.41 - 1.01
April 29 – May 26	0.35	0.22 - 0.57		0.37	0.23 - 0.60		0.38	0.23 - 0.63
May 13 – June 9	0.20	0.12 - 0.34		0.22	0.13 - 0.37		0.22	0.13 - 0.37
May 27 – June 23	0.23	0.14 - 0.39		0.24	0.14 - 0.42		0.26	0.15 - 0.45
June 10 – July 8	0.20	0.12 - 0.34		0.21	0.12 - 0.36		0.21	0.12 - 0.37
June 24 – July 22	0.22	0.13 - 0.38		0.24	0.14 - 0.42		0.25	0.14 - 0.44
July 8 – Aug 5	0.23	0.13 - 0.42		0.25	0.14 - 0.46		0.26	0.14 - 0.47
July 22 – Aug 19	0.23	0.13 - 0.40		0.25	0.15 - 0.44		0.23	0.13 - 0.41
Aug 5 – Sept 2	0.26	0.15 - 0.44		0.26	0.15 - 0.44		0.24	0.14 - 0.42
Aug 19 – Sept 16	0.18	0.10 - 0.31		0.17	0.10 - 0.30		0.18	0.10 -

© 2021 Raifman J et al. *JAMA Network Open.*

								0.32
Sept 2 – Sept 30	0.19	0.11 - 0.34		0.18	0.10 - 0.32		0.19	0.11 - 0.34
Sept 16 – Oct 14	0.14	0.08 - 0.25		0.14	0.08 - 0.24		0.14	0.08 - 0.25
Sept 30 – Oct 27	0.11	0.06 - 0.20		0.11	0.06 - 0.19		0.11	0.06 - 0.20
Oct 14 – Nov 11	0.17	0.10 - 0.31		0.17	0.09 - 0.30		0.17	0.10 - 0.31
**(b) Eating less due to financial constraints**								
**Unemployment insurance**	0.47	0.34 - 0.66						
**Unemployment insurance with CARES $600/week supplement**				0.35	0.23 - 0.53			
**Unemployment insurance without CARES $600/week supplement**				0.57	0.39 - 0.83			
**Unemployment insurance amount**								
No unemployment insurance							Reference	
$1 to 300							0.52	0.31 - 0.86
$301 to 600							0.43	0.23 - 0.80
$601 to 900							0.41	0.23 - 0.76
$901 to 1200							0.44	0.18 - 1.05
$1201 to 1500							0.28	0.08 - 0.92
≥$1500							0.17	0.07 -

								0.40
**Stimulus payment**	0.61	0.40 - 0.91		0.61	0.40 - 0.91		0.62	0.41 - 0.95
**SNAP**	0.90	0.62 - 1.31		0.91	0.63 - 1.31		0.96	0.65 - 1.42
**Currently employed**	0.40	0.30 - 0.51		0.40	0.31 - 0.52		0.39	0.30 - 0.51
**Study wave**								
April 1 – April 28	Reference			Reference			Reference	
April 15 – May 12	0.53	0.35 - 0.81		0.54	0.35 - 0.83		0.55	0.36 - 0.85
April 29 – May 26	0.35	0.22 - 0.56		0.36	0.23 - 0.58		0.38	0.24 - 0.61
May 13 – June 9	0.25	0.15 - 0.41		0.26	0.16 - 0.43		0.27	0.16 - 0.45
May 27 – June 23	0.19	0.11 - 0.33		0.20	0.12 - 0.34		0.20	0.12 - 0.34
June 10 – July 8	0.20	0.12 - 0.34		0.21	0.12 - 0.36		0.21	0.12 - 0.36
June 24 – July 22	0.16	0.09 - 0.28		0.17	0.10 - 0.29		0.17	0.10 - 0.30
July 8 – Aug 5	0.22	0.12 - 0.39		0.24	0.13 - 0.42		0.22	0.12 - 0.40
July 22 – Aug 19	0.24	0.14 - 0.41		0.26	0.15 - 0.44		0.25	0.15 - 0.44

© 2021 Raifman J et al. *JAMA Network Open.*

Aug 5 – Sept 2	0.23	0.13 - 0.38		0.23	0.13 - 0.39		0.22	0. 13 - 0. 38
Aug 19 – Sept 16	0.19	0.11 - 0.34		0.19	0.11 - 0.32		0.20	0. 11 - 0. 35
Sept 2 – Sept 30	0.23	0.14 - 0.40		0.22	0.13 - 0.38		0.21	0. 12 - 0. 37
Sept 16 – Oct 14	0.18	0.10 - 0.31		0.17	0.10 - 0.29		0.17	0. 10 - 0. 30
Sept 30 – Oct 27	0.19	0.11 - 0.33		0.18	0.10 - 0.32		0.18	0. 10 - 0. 31
Oct 14 – Nov 11	0.17	0.09 - 0.29		0.16	0.09 - 0.28		0.17	0. 09 - 0. 30

© 2021 Raifman J et al. *JAMA Network Open.*

eTable 6. Main Difference-in-Differences Estimates of the Association Between Unemployment Insurance and Outcomes of Food Insecurity and Eating Less Among Those Currently Unemployed

	(1) Unemployment insurance			(2) Unemployment insurance and CARES			(3) Unemployment insurance amount	
	Participants: 1104 Observations: 7384			Participants: 1104 Observations: 7384			Participants: 1099 Observations: 6792	
	**Percentage points**	**95% CI**		**Percentage points**	**95% CI**		**Percentage points**	**95% CI**
**(a) Food insecurity**								
**Unemployment insurance**	-5.89	-9.58 - -2.19						
**Unemployment insurance with CARES $600/week supplement**				-7.24	-11.00 - -3.48			
**Unemployment insurance without CARES $600/week supplement**				-4.50	-8.63 - -0.37			
**Unemployment insurance amount**								
No unemployment insurance							Reference	
$1 to 300							-5.53	-10.24 - -0.81
$301 to 600							-3.91	-8.66 - 0.84
$601 to 900							-6.23	-10.40 - -2.05
$901 to 1200							-7.48	-12.57 - -2.39
$1201 to 1500							-6.59	-10.64 - -2.53
≥$1500							-8.35	-12.46 - -4.23

© 2021 Raifman J et al. *JAMA Network Open.*

**Stimulus payment**	-0.89	-4.73 - 2.94		-0.85	-4.70 - 2.99		-0.09	-3.77 - 3.59
**SNAP**	-2.73	-6.76 - 1.31		-2.72	-6.76 - 1.32		-3.32	-7.72 - 1.08
**Study wave**								
April 1 – April 28	Reference			Reference			Reference	
April 15 – May 12	-2.32	-6.31 - 1.68		-2.17	-6.15 - 1.80		-2.27	-6.27 - 1.73
April 29 – May 26	-5.32	-9.78 - -0.87		-5.03	-9.45 - -0.62		-5.50	-9.85 - -1.15
May 13 – June 9	-7.65	-12.51 - -2.78		-7.29	-12.11 - -2.47		-7.83	-12.55 - -3.11
May 27 – June 23	-7.71	-12.57 - -2.85		-7.33	-12.14 - -2.52		-7.63	-12.53 - -2.73
June 10 – July 8	-8.52	-13.50 - -3.54		-8.09	-13.04 - -3.15		-9.03	-13.98 - -4.07
June 24 – July 22	-8.76	-13.84 - -3.68		-8.32	-13.36 - -3.29		-8.57	-13.58 - -3.55
July 8 – Aug 5	-8.66	-13.79 - -3.53		-8.19	-13.26 - -3.11		-8.76	-13.84 - -3.68
July 22 – Aug 19	-7.75	-13.02 - -2.48		-7.36	-12.56 - -2.15		-8.22	-13.62 - -2.82

© 2021 Raifman J et al. *JAMA Network Open.*

Aug 5 – Sept 2	-7.08	-12.45 - -1.71		-7.18	-12.57 - -1.79		-8.21	-13.72 - -2.70
Aug 19 – Sept 16	-9.32	-14.82 - -3.81		-9.92	-15.60 - -4.24		-10.04	-15.66 - -4.43
Sept 2 – Sept 30	-7.49	-12.90 - -2.08		-8.14	-13.72 - -2.57		-8.20	-13.65 - -2.75
Sept 16 – Oct 14	-9.90	-15.50 - -4.30		-10.54	-16.29 - -4.78		-10.61	-16.39 - -4.83
Sept 30 – Oct 27	-11.20	-16.67 - -5.73		-11.85	-17.48 - -6.22		-11.76	-17.37 - -6.15
Oct 14 – Nov 11	-8.25	-13.59 - -2.91		-8.90	-14.41 - -3.39		-8.56	-14.08 - -3.04
**Constant**	24.71	21.40 - 28.03		24.60	21.29 - 27.91		24.12	20.87 - 27.37
**(b) Eating less due to financial constraints**								
**Unemployment insurance**	-7.88	-11.89 - -3.87						
**Unemployment insurance with CARES $600/week supplement**				-9.05	-13.09 - -5.01			
**Unemployment insurance without CARES $600/week supplement**				-6.68	-11.08 - -2.28			
**Unemployment insurance amount**								
No unemployment insurance							Reference	
$1 to 300							-7.15	-12.69 - -1.62

© 2021 Raifman J et al. *JAMA Network Open.*

$301 to 600							-9.39	-14.88 - -3.89
$601 to 900							-8.94	-13.77 - -4.11
$901 to 1200							-7.02	-11.53 - -2.51
$1201 to 1500							-7.92	-12.49 - -3.35
≥$1500							-12.10	-16.99 - -7.21
**Stimulus payment**	-2.38	-6.63 - 1.86		-2.35	-6.60 - 1.91		-1.84	-6.07 - 2.39
**SNAP**	-2.27	-6.61 - 2.07		-2.27	-6.61 - 2.07		-2.82	-7.32 - 1.69
**Study wave**								
April 1 – April 28	Reference			Reference			Reference	
April 15 – May 12	-2.32	-6.31 - 1.68		-2.17	-6.15 - 1.80		-2.27	-6.27 - 1.73
April 29 – May 26	-5.32	-9.78 - -0.87		-5.03	-9.45 - -0.62		-5.50	-9.85 - -1.15
May 13 – June 9	-7.65	-12.51 - -2.78		-7.29	-12.11 - -2.47		-7.83	-12.55 - -3.11

© 2021 Raifman J et al. *JAMA Network Open.*

May 27 – June 23	-7.71	-12.57 - -2.85		-7.33	-12.14 - -2.52		-7.63	-12.53 - -2.73
June 10 – July 8	-8.52	-13.50 - -3.54		-8.09	-13.04 - -3.15		-9.03	-13.98 - -4.07
June 24 – July 22	-8.76	-13.84 - -3.68		-8.32	-13.36 - -3.29		-8.57	-13.58 - -3.55
July 8 – Aug 5	-8.66	-13.79 - -3.53		-8.19	-13.26 - -3.11		-8.76	-13.84 - -3.68
July 22 – Aug 19	-7.75	-13.02 - -2.48		-7.36	-12.56 - -2.15		-8.22	-13.62 - -2.82
Aug 5 – Sept 2	-7.08	-12.45 - -1.71		-7.18	-12.57 - -1.79		-8.21	-13.72 - -2.70
Aug 19 – Sept 16	-9.32	-14.82 - -3.81		-9.92	-15.60 - -4.24		-10.04	-15.66 - -4.43
Sept 2 – Sept 30	-7.49	-12.90 - -2.08		-8.14	-13.72 - -2.57		-8.20	-13.65 - -2.75
Sept 16 – Oct 14	-9.90	-15.50 - -4.30		-10.54	-16.29 - -4.78		-10.61	-16.39 - -4.83
Sept 30 – Oct 27	-11.20	-16.67 - -5.73		-11.85	-17.48 - -6.22		-11.76	-17.37 - -6.15
Oct 14 – Nov 11	-8.25	-13.59 - -2.91		-8.90	-14.41 - -3.39		-8.56	-14.

© 2021 Raifman J et al. *JAMA Network Open.*

								08 - - 3.04
**Constant**	27.12	23.72 - 30.53		27.02	23.61 - 30.43		26.58	23.18 - 29.99

Notes: The sample size was smaller because 15 people were missing a covariate or outcome value in the wave in which they reported not having employment

eTable 7. Main Difference-in-Differences Estimates of the Association Between Unemployment Insurance and Outcomes of Food Insecurity and Eating Less in Very Low-Income Households (<$20 000/Year)

	(1) Unemployment insurance			(2) Unemployment insurance and CARES			(3) Unemployment insurance amount	
	Participants: 282<br>Observations: 2890			Participants: 282<br>Observations: 2890			Participants: 282<br>Observations: 2687	
	**Percentage points**	**95% CI**		**Percentage points**	**95% CI**		**Percentage points**	**95% CI**
**(a) Food insecurity**								
**Unemployment insurance**	-6.58	-12.25 - -0.92						
**Unemployment insurance with CARES $600/week supplement**				-8.07	-14.24 - -1.91			
**Unemployment insurance without CARES $600/week supplement**				-5.58	-11.79 - 0.64			
**Unemployment insurance amount**								
No unemployment insurance								
$1 to 300							-7.15	-14.80 - 0.49
$301 to 600							-3.79	-11.76 - 4.18
$601 to 900							-8.86	-17.15 - -0.58
$901 to 1200							-6.96	-23.99 - 10.06
$1201 to 1500							-10.46	-18.54 - -

© 2021 Raifman J et al. *JAMA Network Open.*

								2.38
≥$1500							-9.07	-15.67 - -2.47
**Stimulus payment**	-5.50	-12.40 - 1.40		-5.51	-12.43 - 1.41		-2.71	-9.86 - 4.43
**SNAP**	-4.22	-10.60 - 2.15		-4.25	-10.64 - 2.15		-2.96	-9.80 - 3.88
**Currently employed**	-6.03	-10.28 - -1.78		-5.88	-10.06 - -1.70		-5.73	-10.09 - -1.37
**Study wave**								
April 1 – April 28	Reference			Reference				
April 15 – May 12	-7.60	-15.42 - 0.22		-7.54	-15.36 - 0.28		-8.41	-16.36 - -0.46
April 29 – May 26	-8.93	-17.57 - -0.30		-8.78	-17.42 - -0.15		-10.77	-19.43 - -2.11
May 13 – June 9	-14.60	-23.67 - -5.53		-14.40	-23.47 - -5.34		-17.13	-26.30 - -7.95
May 27 – June 23	-14.31	-23.44 - -5.17		-14.11	-23.22 - -5.00		-15.88	-25.38 - -6.38
June 10 – July 8	-15.77	-24.86 - -6.69		-15.47	-24.55 - -6.40		-18.24	-27.

© 2021 Raifman J et al. *JAMA Network Open.*

								41 - -9.07
June 24 – July 22	-12.52	-21.55 - -3.49		-12.21	-21.22 - -3.20		-14.87	-24.04 - -5.70
July 8 – Aug 5	-14.11	-23.31 - -4.92		-13.68	-22.92 - -4.45		-15.59	-25.13 - -6.05
July 22 – Aug 19	-14.59	-24.19 - -4.99		-14.21	-23.82 - -4.60		-16.79	-26.83 - -6.76
Aug 5 – Sept 2	-13.87	-23.06 - -4.68		-13.81	-23.01 - -4.60		-16.54	-25.99 - -7.09
Aug 19 – Sept 16	-16.59	-26.27 - -6.90		-16.86	-26.64 - -7.09		-19.50	-29.51 - -9.48
Sept 2 – Sept 30	-16.02	-25.64 - -6.39		-16.30	-25.97 - -6.63		-18.12	-27.94 - -8.29
Sept 16 – Oct 14	-18.68	-28.36 - -8.99		-18.97	-28.70 - -9.24		-21.05	-31.01 - -11.09
Sept 30 – Oct 27	-19.80	-29.23 - -10.38		-20.09	-29.56 - -10.62		-21.80	-31.49 - -12.11

© 2021 Raifman J et al. *JAMA Network Open.*

-16.36		-25.98 - -6.74		-16.66	-26.34 - -6.99		-18.25	-28.31 - -8.18
**Constant**	44.26	37.43 - 51.09		44.19	37.34 - 51.05		43.93	37.05 - 50.80
**(b) Eating less due to financial constraints**								
**Unemployment insurance**	-8.88	-16.15 - -1.61						
**Unemployment insurance with CARES $600/week supplement**				-9.49	-17.19 - -1.78			
**Unemployment insurance without CARES $600/week supplement**				-8.20	-15.63 - -0.76			
**Unemployment insurance amount**								
No unemployment insurance								
$1 to 300							-9.82	-19.92 - 0.27
$301 to 600							-6.21	-17.05 - 4.63
$601 to 900							-13.55	-24.70 - -2.41
$901 to 1200							-11.31	-18.69 - -3.93
$1201 to 1500							-9.97	-18.60 - -1.35

© 2021 Raifman J et al. *JAMA Network Open.*

≥$1500							-12.17	-23.00 - -1.34
**Stimulus payment**	-2.38	-9.41 - 4.65		-2.39	-9.44 - 4.66		0.18	-6.97 - 7.33
**SNAP**	-4.72	-11.95 - 2.52		-4.76	-11.99 - 2.47		-4.12	-11.55 - 3.31
**Currently employed**	-6.64	-11.68 - -1.59		-6.42	-11.39 - -1.46		-6.34	-11.47 - -1.21
**Study wave**								
April 1 – April 28	Reference			Reference				
April 15 – May 12	-10.16	-17.30 - -3.03		-10.14	-17.28 - -2.99		-10.78	-18.06 - -3.50
April 29 – May 26	-15.78	-23.93 - -7.64		-15.72	-23.88 - -7.56		-17.06	-25.46 - -8.66
May 13 – June 9	-15.53	-24.27 - -6.78		-15.46	-24.19 - -6.73		-17.44	-26.41 - -8.47
May 27 – June 23	-19.58	-28.71 - -10.45		-19.52	-28.63 - -10.41		-22.20	-31.32 - -13.07
June 10 – July 8	-19.97	-28.98 - -10.96		-19.77	-28.81 - -10.74		-22.14	-31.29

© 2021 Raifman J et al. *JAMA Network Open.*

								- -13.00
June 24 – July 22	-19.56	-28.58 - -10.54		-19.39	-28.41 - -10.36		-21.35	-30.54 - -12.16
July 8 – Aug 5	-21.62	-30.86 - -12.39		-21.38	-30.63 - -12.13		-23.63	-33.07 - -14.19
July 22 – Aug 19	-22.06	-31.22 - -12.90		-21.81	-30.96 - -12.65		-23.92	-33.23 - -14.62
Aug 5 – Sept 2	-21.27	-30.57 - -11.97		-21.21	-30.53 - -11.89		-22.94	-32.52 - -13.35
Aug 19 – Sept 16	-21.61	-31.03 - -12.19		-21.81	-31.24 - -12.38		-23.49	-33.12 - -13.86
Sept 2 – Sept 30	-22.05	-31.76 - -12.34		-22.27	-32.00 - -12.53		-24.70	-34.74 - -14.66
Sept 16 – Oct 14	-23.45	-32.80 - -14.10		-23.66	-33.03 - -14.29		-25.94	-35.49 - -16.39
Sept 30 – Oct 27	-21.92	-31.67 - -12.18		-22.13	-31.89 - -12.38		-24.35	-34.36 - -14.35

© 2021 Raifman J et al. *JAMA Network Open.*

Oct 14 – Nov 11	-23.88	-32.97 - -14.79		-24.10	-33.23 - - 14.98		-25.57	- 34. 90 - - 16. 25
**Constant**	46.72	39.96 - 53.47		46.65	39.88 - 53.42		46.56	39. 77 - 53. 35

© 2021 Raifman J et al. *JAMA Network Open.*
